# Supplementary material for: Molecular Dynamic Simulation Analysis of a Novel Missense Variant in CYB5R3 Gene in Patients with Methemoglobinemia
Source: Medicina (Kaunas). 2023 Feb 16;59(2):379. doi: 10.3390/medicina59020379 (PMC9967277; doi:10.3390/medicina59020379)
Supplement: Supplementary file 1 [file medicina-59-00379-s001.zip › medicina-2096565-supplementary.pdf]

Supplementary Table 1: **(A & B)** Pathogenicity prediction and scores of the identified missense variant in *CYB5R3* by different prediction tools.

(A) Individual Predictions

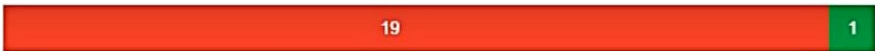

Prediction Tool	Prediction	Score	Rankscore
BayesDel addAF *dbNSFP* version 4.2	Damaging	0.2351	0.772
BayesDel noAF *dbNSFP* version 4.2	Damaging	0.09994	0.769
CADD		26.5	
DANN	0.9888		
DEOGEN2 *dbNSFP* version 4.2	Damaging	0.6057	0.8743
EIGEN *dbNSFP* version 4.2	Pathogenic	0.608	0.7368
EIGEN PC *dbNSFP* version 4.2	Pathogenic	0.4883	0.6721
FATHMM *dbNSFP* version 4.2	Damaging	-2.27	0.8743
FATHMM-MKL *dbNSFP* version 4.2	Damaging	0.9342	0.5817
FATHMM-XF *dbNSFP* version 4.2	Damaging	0.5927	0.5883
LIST-52 *dbNSFPversion* 4.2	Damaging	0.9751	0.912
LRT *dbNSFP* version 4.2	Deleterious	0.00003999	0.6293
M-CAP *dbNSFP* version 4.1	Damaging	0.3817	0.9299
MVP *dbNSFP* version 4.2	Pathogenic	0.9753	0.975
MutPred *dbNSFP* version 4.1	Pathogenic	0.687	0.8245
Mutation assessor *dbNSFP* version 4.1	High	4.29	0.9822
MutationTaster *dbNSFP* version 4.1	Disease causing		0.81
PROVEAN *dbNSFP* version 4.1	Damaging	-3.83	0.7212
SIFT *dbNSFP* version 4.2	Damaging	0.001	0.7849
SIFT4 *DbNSFP* version 4.1	Damaging	0.002, 0.001	0.8335

(B) Pathogenicity Scores

Meta Scores

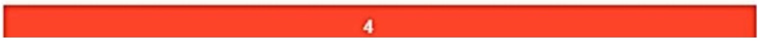

MetaLR *dbNSFP* version 4.1	Damaging	0.7903	0.9289
MetaSVM *dbNSFP* version 4.2	Damaging	0.7523	0.9382
MetaRNN *dbNSFP* version 4.1	Damaging	0.9036	0.8972
REVEL *dbNSFP* version 4.2	Pathogenic	0.8149	0.9413
